# The Physiological and Psychological Benefits of Dance and its Effects on Children and Adolescents: A Systematic Review

**DOI:** 10.3389/fphys.2022.925958

**Published:** 2022-06-13

**Authors:** Dan Tao, Yang Gao, Alistair Cole, Julien S. Baker, Yaodong Gu, Rashmi Supriya, Tomas K. Tong, Qiuli Hu, Roger Awan-Scully

**Affiliations:** ^1^ Faculty of Sport Science, Ningbo University, Zhejiang, China; ^2^ Department of Government and International Studies, Hong Kong Baptist University, Hong Kong, China; ^3^ Department of Sport Physical Education and Health, Centre for Health and Exercise Science Research, Hong Kong Baptist University, Hong Kong, China

**Keywords:** children, adolescent, dance intervention, dance therapy, physical activity, health policy and practice

## Abstract

**Background:** The aim of this review was to examine the physiological and psychological benefits of dance and its effects on children and adolescents. We consider the therapeutic benefits of dance and outline the potential of dance as an alternative therapy for certain pathologies and medical disorders. Secondly, we summarize the types of dances used in physical interventions, and comment on the methodologies used. Finally, we consider the use of dance as a different exercise modality that may have benefits for increased physical activity generally, and for increased physical education provision in schools.

**Methods:** A structured search strategy was conducted using the databases of PubMed, MEDLINE, Web of science, PsycARTICLES, and Social Science database. This review used the Preferred Reporting Items for Systematic Reviews and Meta-Analyses (PRISMA) guidelines for systematic reviews. Studies that were published in the past 20 years were considered for inclusion. All written publications were searched for in English, and all articles included in this review were peer reviewed full papers.

**Conclusion:** The key findings from this review indicate that dance is a feasible alternative to traditional physical activity. The findings also indicate that dance provides physiological and psychological benefits to healthy and medically compromised populations. Implementation of dance programs in schools and society generally needs serious consideration by policy makers. We hope that the results of this review stimulate debate and provide the necessary evidence to profile dance as a viable alternative medium of physical activity. Comprehensive and integrated changes will be needed including economical and legislative support from politicians and associated governmental agencies. The findings reported here are important and have implications for health policy change, reconfiguration, and implementation.

## 1 Introduction

Physical Activity (PA) provides positive health benefits. The benefits include increases in cardiovascular fitness, physiological and psychological health, and musculoskeletal strength. In addition, PA has been successful in the prevention and treatment of diseases such as stroke, diabetic problems, high blood pressure, and certain cancers. PA has also been proven to be beneficial for maintaining a healthy body weight, enhancing quality of life, and contributing to individual well-being (WHO, 2020). PA also contributes positively in influencing social connectedness (Duberg et al., 2020). Equally, a decline in PA or lack of engagement, is one of the major risk factors associated with good health and mortality. Individuals not engaging in PA are prone to a 20%–30% risk of death increase compared to individuals participating in PA (WHO, 2020).

It has also been reported that engagement in regular PA is essential for healthy growth and development in children (WHO, 2020). The growth and developmental period in young people, is a time when negative social, and psychological experiences can affect cognitive, intellectual, and rational development (Lund et al., 2018). In support of this, most preventive strategies have increased success rates when the focus of the preventive strategy occurs in the early years and decades of life (Kieling et al., 2011). The World Health Organization (WHO) suggests that young people aged 5–17 years should participate in on average 60 min a day of moderate-to-vigorous exercise. The exercise type should mostly include aerobic activity executed over a 7-day period. The inclusion of high intensity performances, such as strength exercises, for at least 3 days a week is also desirable. The time spent participating in sedentary activities, particularly television and computer screen time, also needs to be minimized (WHO, 2020).

However, despite this, 80% of the world’s adolescent population do not participate in physical activity (WHO, 2020). This figure is particularly alarming in female populations. One reason for lack of participation by females could be related to physical development. As females grow and develop, they become more aware of the significance of femininity, and involvement in exercise is often depicted as not corresponding to this image (Slater and Tiggemann, 2010). This problem has become even more acute during the COVID-19 pandemic. Quarantine stratagems have had a poor impact on PA. Research has revealed significant decreases in PA during this period (Tao et al., 2021). These undesirable health consequences of quarantine measures, that include psychological stress and greater physical inactivity, need consideration post quarantine to promote increased physical activity and associated health benefits (Füzéki et al., 2020).

Dance movement practice (DMP) is a type of art therapy that has been entrenched in modern culture for 70 years. Dance provides benefits for participants that are both personal and independent. Dance participation also provides physical and mental wellbeing (Tao et al., 2021). Further benefits include defining and consolidating body image; illuminating the ego; providing relief of physical tension, anxiety, and aggression, while decreasing cognitive and kinesthetic confusion. Dance also increases the capacity for interaction, increases pleasure, fun, and impulsiveness (Jeong et al., 2005). In addition, children subjected to emotional illness have certain emotional and physical limitations when engaging in traditional PA. Dance is a physical activity medium that can provide discrete and precise exercise prescriptions for these individuals.

Research related to dance interventions has demonstrated a rising trajectory in recent years. However, dance still needs to be recognized as viable physical activity alternative. In earlier reviews on children and adolescent populations, it was demonstrated that dance therapy could promote beneficial health aspects in children with autism spectrum disorders (Aithal et al., 2021). The research outlined that dance may be associated with positive physical, cognitive and sociological adaptations for children with emotional and physical problems, however, the selection of articles used in the study were of a poor quality and need to be viewed with caution (May et al., 2021). There are a further three articles focusing on the association between dance, well-being and health, however, there are some imperfections in the studies. These include not fully exploring the outcomes of the dance intervention including other types of PA (Mansfield et al., 2018); less coverage for age groups (Carson et al., 2017). In addition, some studies only verified the amount of time spent performing at moderate to vigorous intensities in children and adolescents during the dance class. Further studies need to expand on the potential benefits and exercise intensities and durations used in these groups (Dos Santos et al., 2021). To the best our knowledge, there are no existing studies that have explored fully the benefits of dance interventions for children and adolescents. Further research is required to systematically report on all aspects related to the benefits of dance as a viable physical activity for this population. Therefore, the purpose of this review was to select all the studies utilizing a dance intervention in children and adolescents over the past 20 years; examine the dance intervention method; verify the outcomes; summarize the strengths and limitations of the research; and to provide evidence that dance can be used for children and adolescents as a suitable and viable physical activity in the future.

The four main objectives of this systematic review were to examine: 1) The emotional and physical benefits of dance in children and adolescents; 2) To consider the benefits of dance as an alternative physical activity/therapy for children and adolescents with certain medical disorders; 3) To examine the types of dances selected for the interventions reviewed, and the specific training loads required. This information may be useful for future research and implementation; 4) To consider dance as an alternative PA for school physical education provision.

## 2 Methodology

### 2.1 Eligibility Criteria

Studies focusing on the use of dance as an intervention and studies that involved children and adolescents inclusive of up to 18 years of age were included. Studies that were written in English and published in the past 20 years were considered. Meta-analyses or systematic review/review articles and pilot studies were excluded. Studies that used professional/semi-professional dancers as participants were also excluded. For inclusion in this review, each selected article must have been subjected to a peer review process prior to publication. In addition, the article had to present a clear, consistent methodology.

### 2.2 Information Sources and Search Strategy

A literature search was completed on 25 November 2021, articles were found by examining electronic databases to locate research studies that focused on the use of dance as an intervention for children and adolescents. The search methodology used in this study was based on the PICOS system (Jensen, 2017) and followed the Preferred Reporting Items for Systematic Reviews and Meta-Analyses (PRISMA) guidelines (Moher et al., 2009). PROSPERO Registration Number is CRD42022326748. To locate articles for inclusion in this review the databases of PubMed, MEDLINE, Web of science, PsycARTICLES, and Social Science databases were comprehensively searched. Publications were identified for inclusion using the MeSH terms Children OR Teenager OR Adolescent OR Schoolchildren OR Student AND Dance OR Dancing OR Ballroom-dance OR Sport-dance OR Ballet OR Jazz OR Folk-dance OR Hip-Pop OR Square-dance OR Dance-movement-therapy OR Dance-effectiveness OR Dance-interventions. Additionally, other review or systematic review articles were used as guidelines to source articles that matched the inclusion criteria (Sheppard and Broughton, 2020).

### 2.3 Study Selection and Data Collection Process

**FIGURE 1:**
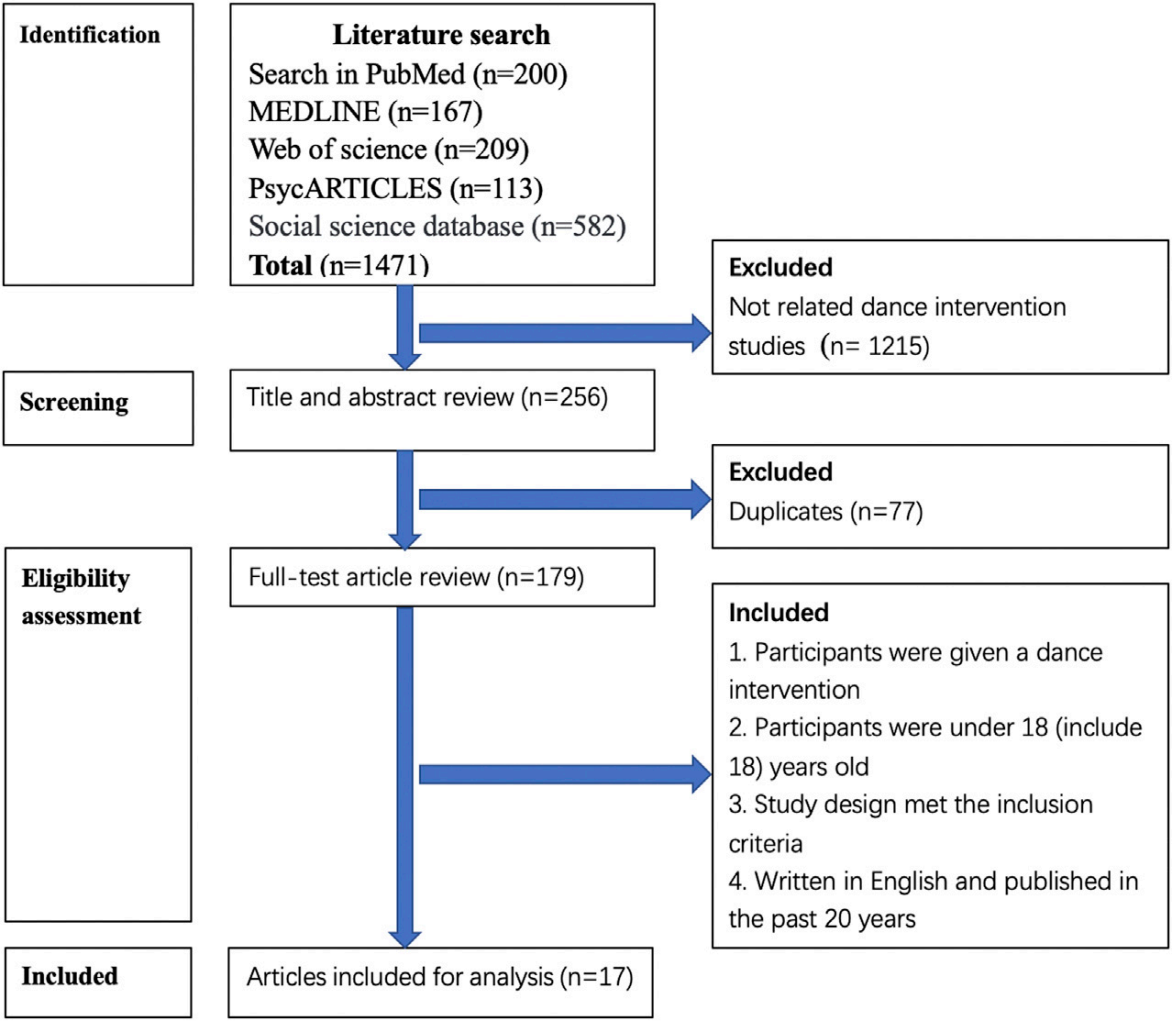
Flowchart: Article selection process.

Articles used in this review were selected by identification of the search terms contained in the full texts. Articles not meeting the inclusion criteria or meeting the exclusion criteria were discarded. Figure 1 represents a flowchart of the process of identification and selection of relevant studies. The study selection process was confirmed by two authors (DT and JSB). If there was a disagreement between the two authors in the selection process, a third author (RS) contributed to resolving any article selection or exclusion issues.

Data extraction sheets were then developed. The first author (DT) extracted the data from included studies and the second author (JSB) checked the extracted articles. Any disagreements between authors was resolved by amicable discussion; if no consensus was accomplished, a third author (RS) decided the outcome. The following information for each study was extracted: 1) The citation information; 2) Participants demographics; 3) Dance intervention; 4) Study design/Measurements/Type of data; 5) Key findings.

### 2.4 Risk of Bias for Individual Studies

Risk of bias variables included random sequence generation, allocation concealment, blinding of patients and personnel, blinding of outcome assessment, incomplete outcome data, selective outcome reporting and other bias was examined following the Cochrane collaboration Risk of Bias Tool (Higgins and Altman, 2017; Higgins et al., 2011). 15 RCT studies were divided into three categories, low risk, high risk, or unclear risk (when a study reported inadequate information to rate a specific domain). Risk of bias was also assessed separately using Review Manager 5.4.1 software. This assessment was completed by DT and RS independently; any disagreements on the risk of bias were adjudicated by JSB.

## 3 Results

### 3.1 Study Selection and Characteristics

In total, 179 articles, after excluding duplicates, were identified by the literature search process. Following the inclusion and exclusion criteria, 162 were discarded resulting in 17 remaining articles (see Figure 1). Included articles were summarized into tables (see Table 1 and Table 2 for further details). Ten (59%) of the 17 studies recruited females as participants (Jeong et al., 2005; Robinson et al., 2010; O’Neill et al., 2011; Wagener et al., 2012; Duberg et al., 2013; Staiano et al., 2017b; Staiano et al., 2017a; Duberg et al., 2020; Sandberg et al., 2021; Högström et al., 2022), the remaining seven articles were studies inclusive of both genders (Morris et al., 2013; Anjos and Ferraro, 2018; Bollimbala et al., 2019; Oppici et al., 2020; Goswami et al., 2021; Raghupathy et al., 2021; Rudd et al., 2021). There were 15 (88%) studies that used randomized controlled trials (RCT) (Jeong et al., 2005; Robinson et al., 2010; Wagener et al., 2012; Duberg et al., 2013; Staiano et al., 2017b; Staiano et al., 2017a; Anjos and Ferraro, 2018; Bollimbala et al., 2019; Duberg et al., 2020; Oppici et al., 2020; Goswami et al., 2021; Raghupathy et al., 2021; Rudd et al., 2021; Sandberg et al., 2021; Högström et al., 2022), and 8 (47%) studies used both quantitative and qualitative mixed methods to collect data (Jeong et al., 2005; Robinson et al., 2010; O’Neill et al., 2011; Wagener et al., 2012; Duberg et al., 2013; Morris et al., 2013; Staiano et al., 2017a; Goswami et al., 2021). Included studies examined objective indicators and self-reported measurements with physiological (41%) (O’Neill et al., 2011; Morris et al., 2013; Staiano et al., 2017b; Staiano et al., 2017a; Anjos and Ferraro, 2018; Sandberg et al., 2021; Högström et al., 2022). psychological (47%) (Jeong et al., 2005; Robinson et al., 2010; Wagener et al., 2012; Duberg et al., 2013; Bollimbala et al., 2019; Duberg et al., 2020; Oppici et al., 2020; Rudd et al., 2021) and medical (12%) (Goswami et al., 2021; Raghupathy et al., 2021) included as the three main aspects of this study. The results and key concepts of the review are discussed below.

**TABLE 1:** Summary of participant age groups, research design, methodological approach and outcome examined.

	Dance intervention type		Research design			Methodological approach			Outcome examined			
Gender group	Choreographed	Other type	RCT	Non-RCT	Cross-sectional	Quantitative	Qualitative	Mix	Physiological	Psychological	Medical	Total studies for gender group
Female	1	9	9		1	1	3	6	5	5		10
Both Gender	4	3	6	1		5		2	2	3	2	7
Total	5	12	15	1	1	6	3	8	7	8	2	17

Other type in the dance intervention part = Exergaming, African dance, Jazz dance, street, Contemporary dance, Traditional India dance, Folk dance, India classical dance, Hip-pop, Step dance, Educational dance, Dance-based PE, Dance and Yoga.

**TABLE 2:** Detailed summary of the study details.

Citations	Participant demographics	Dance interventions	Study design/Measurements/Type of data	Key findings
Wagener et al. (2012)	*n* = 40 Female Age 12–18 years old	Exergaming (video game dance)	RCT	Positive impact of dance-based exergaming on obese adolescents’ psychological functioning and perceived competence to continue exercise
	Obese adolescents		1. BMI 2. Perceived Competence Scale (PCS) 2. The Behavior Assessment System for Children-2 (BASC-2) 3. Parent Rating Scales-Adolescent version (PRS-A) 4. Adolescent Self-Report Scales (SRP-A)	
	United States		Quantitative and Qualitative	
Sandberg et al. (2021)	*n* = 112 Female	African dance, different choreographies to popular music in the show/jazz dance, street and contemporary dance genre	RCT	1. Dance intervention can be effective in decreasing daytime tiredness
	Age 13–18 years old < Participants with stress-related mental health problems		Pittsburgh Sleep Quality Index	2. Nonpharmacological interventions to decrease stress-related problems among adolescents
			Qualitative	
Rudd et al. (2021)	*n* = 55 Both gender	Specially choreographed dance routine	RCT	1. Dance intervention improved inhibitory control and potentially working memory capacity
	Age 6–7 years old		1. Executive functions (working memory capacity, cognitive flexibility and inhibitory control)	2. Dance intervention did not improve motor competence beyond typical development
	Primary school student		2. Motor competence	
	Australia		Quantitative	
Raghupathy et al. (2021)	*n* = 36 Both gender	Traditional India dance	RCT	1. The traditional Indian dance improved the locomotor skills of children with Down syndrome than that of neuromuscular exercises
	Age 6–10 years old		1. Test of Gross Motor Development–2 (TGMD–2) 2. Four Square Step Test (FSST) 3. Pediatric balance scale	2. Both the dance and neuromuscular training equally impacted the balance capacity
	Children with DS		Quantitative	
	India			
Morris et al. (2013)	*n* = 378 Both gender	Specially choreographed dance routine	A non-RCT	1. Significant increases in physical activity, endurance fitness and a reduction in the rate of increase in sum of skinfolds
	Age 9.75 ± 0.82 years old		1. Physical activity 2. Food intake 3. Anthropometric measure 4. Knowledge of healthy lifestyles 5. Psychological measures	2. There was no intervention effect on any of the dietary variables, knowledge, and the majority of psychological variables
	Primary school student		Quantitative and Qualitative	
	United Kingdom			
Jeong et al. (2005)	*n* = 40 Female	Specially choreographed dance routine	RCT	Dance movement therapy improved the negative psychological symptoms and modulated serotonin and dopamine concentrations in adolescent girls with mild depression
	Age 16 years oldMiddle school student with depression		1. Measurement of Psychological Distress (SCL-90-R) 2. Measurements of Neurohormones	
	Korea		Quantitative and Qualitative	
Bollimbala et al. (2019)	*n* = 34 Both gender	Folk dance	RCT 1. Convergent thinking 2. Divergent thinking	1. Dance intervention improved convergent thinking 2. Participants with normal BMI improved in two divergent thinking components 3. Not permit us to establish a causal relationship between PA and the development of creative potential
	Age 12 years old	Specially choreographed dance routine	Quantitative	
	Primary school students			
	India			
Staiano et al. (2017b)	*n* = 41 Female	Exergaming (video game dance)	RCT	Exergaming reduced body fat and increased BMD
	Age 14–18 years old		1. Physical examination and electrocardiogram 2. Anthropometry 3. Blood pressure 4. Body composition	
	Overweight and obese girls		Quantitative	
Robinson et al. (2010)	*n* = 261 Female	Hip-hop	RCT	1.Not significantly reduce BMI gain compared with health education 2. Potentially reductions in lipid levels, hyperinsulinemia, and depressive symptoms
	Age 8–10 years old	African dance	1. Body mass index (BMI) 2. Waist circumference, Triceps skinfold thickness, resting blood pressure and heart rate 3, Fasting serum insulin, glucose, lipid levels 4. Physical activity level 5. Television viewing, videotape viewing, video game and computer use 6. self-reported psychosocial measures Quantitative and Qualitative	
	African American or black girls	Step dance		
Duberg et al. (2013)	*n* = 59 Female	African dance	RCT	1. Improve self-rated health for adolescent girls with internalizing problems 2. The improvement remained a year after the intervention
	Age 13–18 years old	Jazz	1. Self-rated health 2. Adherence to and experience of the intervention	
	Participants with stress and psychosomatic symptoms	Contemporary dance	Quantitative and Qualitative	
	Swedish			
Duberg et al. (2020)	*n* = 112 Female	African dance	RCT	1. Dance interventions may reduce somatic symptoms and emotional distress in adolescent girls 2. May constitute a nonpharmacological complement to school health services
	Age 13–18 years old	Jazz	Questionnaires with somatic symptoms and emotional distress	
	Participants with stress-related somatic symptoms and emotional distress	Street dance	Qualitative	
	Swedish			
Isabelle de et al. (2018)	*n* = 85 Both gender	Educational dance	RCT	Educational dance helped the children’s motor development
	Elementary school student		Motor developments	
	Brazil		Quantitative	
Staiano et al. (2017a)	*n* = 37 Female	Exergaming (video game dance)	RCT	Positive impacts on adolescent girls’ self-reported PA, television viewing, self-efficacy, and intrinsic motivation
	Age 14–18 years old		1. Anthropometric measurements 2. Physical activity level 3. Behavioral observation 4. Self-report survey	
	Participants with overweight or obese		Quantitative and Qualitative	
	United States			
O'Neill et al. (2011)	*n* = 149 Female Age 11–18 years old	Ballet	Cross-sectional design	Dance classes can make an important contribution to girls’ total physical activity
	Dance studios girls	Jazz	1. Anthropometric measurements 2. Physical activity level 3. Self- report surveyQuantitative and Qualitative	
	United States	Tap dance		
Oppici et al. (2020)	*n* = 80 Both gender	Jazz-dance choreography	RCT 1. Working memory capacity 2. Motor competence 3. Cognitive flexibility and inhibitory control	1. Dance practice coupled with a high cognitive challenge could improve working memory capacity and motor competence in children 2. The difference between groups was not statistically significant
	Age 8.8 ± 0.7 years old		Quantitative	
	Primary school children			
	Australia			
Högström et al. (2022)	*n* = 112 Female	Dance and Yoga	RCT	Significantly greater pain reduction
	Aged 9–13 years old		Self-report 1. Maximum abdominal pain 2. bases and related information	
	Diagnosed with FAP or IBS with persistent pain		Qualitative	
	Sweden			
Jyotindea et al. (2021)	*n* = 59 Both gender	Specially choreographed dance routine	RCT	Home-centered activity-based therapy is a feasible and practical modality of CP rehabilitation
	Age 5–12 years old		1.6-minute-walk-test 2.10-minute-fast-walk-test 3. Ashworth scale (MAS) 4. Tardieu scale (MTS) 5. Gross Motor Function Classification System (GMFCS) 6. Gross Motor Function Measure-88 (GMFM-88) 7. Cerebral Palsy Quality of Life (CP-QoL)	
	Participants with spastic diplegic CP		Quantitative and Qualitative	

### 3.2 Risk of Bias Within Individual Studies

**FIGURE 2:**
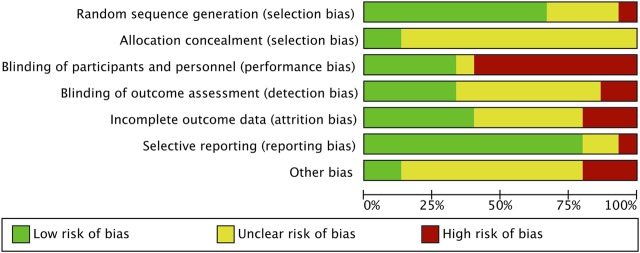
The summary of risk of bias.

**FIGURE 3:**
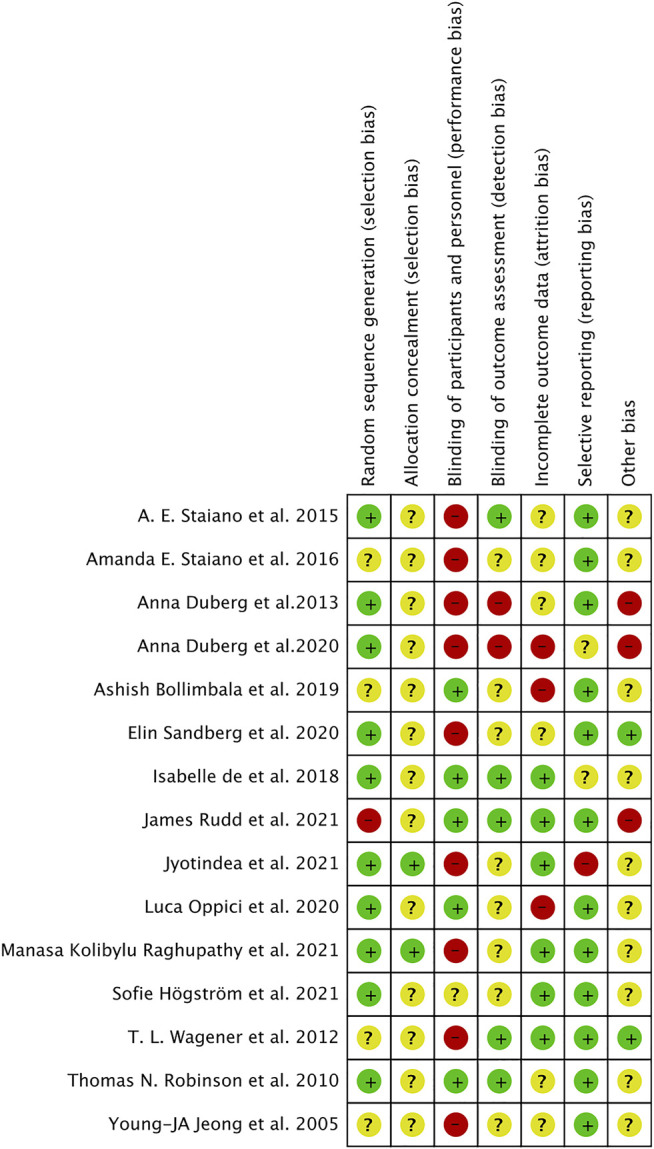
Risk of bias for each study.

A summary of the risk of bias assessment is shown in Figure 2. Each study is outlined in Figure 3. According to the assessment criteria no studies were rated as being of low risk of bias. The primary reason for a high risk of bias was the lack of participant and personnel blinding (60%) across the majority of studies; other reasons were incomplete outcome data (20%) and other bias (20%) (the authors explained in the risk factors that may influence the results of the study) separately. Selective reporting (80%) and random sequence generation (67%) items in most studies were rated as low risk of bias, and most studies rated as being unclear risk of bias due to lack of clear reporting in allocation concealment (87%), other bias included (67%) and blinding of outcome assessment (53%) items.

### 3.3 Dance Selection

There is no consensus regarding the dance intervention type or intervention duration period in the existing literature. The ideal intervention would include different dance types for matching different participants (gender, religion, etc.). During the intervention, teaching supportively and non-judgmentally were important. A further important factor for consideration during dance implementation studies was cultural diversity. Certain traditional or special dances for certain areas and populations may demonstrate greater participation and better intervention performances and results. For further information see Table 2.

In relation to the articles selected for this review, they mainly included African dance (Robinson et al., 2010; Duberg et al., 2013; Duberg et al., 2020; Sandberg et al., 2021), Jazz (O’Neill et al., 2011; Duberg et al., 2013; Duberg et al., 2020; Oppici et al., 2020; Sandberg et al., 2021), Contemporary dance (Duberg et al., 2013; Sandberg et al., 2021), Exergaming video dance (Wagener et al., 2012; Staiano et al., 2017b; Staiano et al., 2017a), Ballet (O’Neill et al., 2011), Jazz dance, Tap dance (O’Neill et al., 2011), Street dance (Duberg et al., 2020; Sandberg et al., 2021), Hip-pop (Robinson et al., 2010), Step dance (Robinson et al., 2010), Fork dance (Bollimbala et al., 2019), Traditional Indian dance (Raghupathy et al., 2021), Education dance (Anjos and Ferraro, 2018), Dance combined with Yoga (Högström et al., 2022), and specially choreographed dance routine (Jeong et al., 2005; Morris et al., 2013; Bollimbala et al., 2019; Goswami et al., 2021; Rudd et al., 2021).

For ethical reasons, control groups should be offered dance interventions following completion of the studies. Researchers should ensure professional choreography of dance interventions and make the routines both physically intense and enjoyable. Researchers should also consider the acceptability of dance for males in the process of wide-ranging dance promotion.

### 3.4 Intervention Monitoring

It is very important in dance study design to monitor intervention training loads. During dance interventions, setting a related exercise target Heart Rate (HR) to ensure that participants reach a predetermined level of exercise is essential. Depending on physical fitness levels, population groups, and ability, variations in intensity of exercise including high-intensity exercise or moderate to vigorous exercise may be used. The intervention duration should be longer than the time required for habit-forming at least to allow participants to continue dancing following the intervention. This important methodological issue has been neglected in previous studies. Only certain articles mentioned intervention monitoring, such as the use of Heart Rate (Wagener et al., 2012), and the Borg Rating of Perceived Exertion (RPE) (Borg 1998). However, scientific and professional monitoring of training intensities is lacking; experimental design and interventions are needed that are based on strong scientific evidence or follow the WHO guidelines (WHO, 2020).

### 3.5 Outcome Measure Summary

Outcome measures outlined in this review include objective measurement methods and self-rated measures of activity. We suggest that a combination of these two measurement methodologies will provide a more complete understanding of the participants’ responses to the intervention results based on desired outcome measures (See Table 2).

Of the articles selected for this review, articles included objective measurements, such as anthropometric measurements (Robinson et al., 2010; O’Neill et al., 2011; Staiano et al., 2017a; Staiano et al., 2017b), physical activity levels (Robinson et al., 2010; O’Neill et al., 2011; Morris et al., 2013), heart rate (HR) (Robinson et al., 2010; Robinson et al., 2010; O’Neill et al., 2011; Staiano et al., 2017b), body mass index (BMI) (Robinson et al., 2010; Morris et al., 2013; Staiano et al., 2017b), blood pressure (BP) (Högström et al., 2022), blood samples for total cholesterol, triglycerides, glucose, insulin and high-density lipoprotein (HDL)-cholesterol, low-density lipoprotein (LOD)-cholesterol, body composition (Robinson et al., 2010; Staiano et al., 2017b), Four-Square Step Test (FSST) (Raghupathy et al., 2021), Test of Gross Motor Development-2 (TGMD-2) (Raghupathy et al., 2021), plasma serotonin and dopamine concentrations (Jeong et al., 2005), 6-minute-walk-test, 10-minute-fast-walk-test (Goswami et al., 2021), executive functions (Oppici et al., 2020; Rudd et al., 2021), motor development (Anjos and Ferraro, 2018; Goswami et al., 2021; Raghupathy et al., 2021).

Questionnaire measurements, included the Perceived Competence Scale (PCS) (Wagener et al., 2012), Adolescent Self-Report Scales (SRP-A) (Wagener et al., 2012), Measure of Psychological Distress (SCL-90-R) (Jeong et al., 2005), Pittsburgh Sleep Quality Index (Sandberg et al., 2021), the scale for Self-efficacy for Physical Activity, the scale for Self-efficacy for Healthy Eating (Morris et al., 2013), Symptom Check List-90-Revison (SCL-90-R), Child Behavior Checklist (Oppici et al., 2020), McKnight Risk Factor Survey; Female African American Pre-adolescent Body Figure Silhouettes; 10-item short form of the Children’s Depression Inventory; 10-item Rosenberg Self-Esteem scale (Robinson et al., 2010), Godin-Shephard Leisure Time PA, Intrinsic Motivation Inventory to assess their enjoyment and experience of playing exergames (Staiano et al., 2017a).

Outcome measures also included measures derived from the authors, such as participants-reported competency regarding maintaining regular exercise, internalizing and externalizing symptomatology, social stress, relationship with parents, interpersonal relationships, social skill and pro-social behaviors (Wagener et al., 2012), knowledge of healthy lifestyles test (Morris et al., 2013), questions regarding lifestyle, self-rated health, emotional distress, psychosomatic symptoms, feelings, depression, sleep, school, interests, friends, leisure time, and how the subjects enjoyed dance (Duberg et al., 2013), maximum abdominal pain (Högström et al., 2022), somatic symptoms and emotional distress (Duberg et al., 2020), executive functions, working memory capacity, cognitive flexibility, inhibitory control, and motor competence (Rudd et al., 2021).

## 4 Discussion

### 4.1 Physiological Benefits of Dance

#### 4.1.1 Dance Intervention Contributed to Access to Physical Activity

An acceptable exercise should be enjoyable, fun, safe and make the participants feel elated. The high participation rate and ease of acceptance and performance made dance interventions a sustainable and flexible alternative mediator to increase physical activity. Dance intervention programs can be performed in safe community spaces, free of charge. This provides a good opportunity for the parents to have more communication and social interaction with their children while facilitating intergenerational togetherness. These are good social outcomes for parental involvement with children in addition to providing a good family exercise environment (Morris et al., 2013). Previously, a dance study enrolled 149 girls (11–18 years-old) into dance intervention group. Activity was performed using structured dance classes in a dance studio. Dancing occupied 29 percent of the individual’s moderate-to vigorous-physical activity (MVPA) (within 1 week). During intervention days the female participants were 70% more MVPA than non-program time (O’Neill et al., 2011).

#### 4.1.2 Physical Fitness Improvement

Young people aged between 15 and 24 years encounter greater daytime fatigue than other age groups; this problem seems to be more severe among girls. Daytime tiredness increases in adolescents with health problems, these include sleep disturbances, and mental health issues. These associated psychological issues, somatic problems, and negative attitudes towards life decrease school achievement and satisfaction (Sandberg et al., 2021). An article investigating 8 months dance intervention, using a total of 48 classes over 24 weeks (except holidays), found that daytime fatigue significantly decreased in a dance intervention cohort at 8 months (*p* = 0.024). Follow up measures observed that there were still decreases at 12- and 20-months post intervention separately. The quality of sleep indicators also improved during the dance intervention. These included, falling asleep (*p* = 0.0037), less worried sleep (*p* = 0.041), and waking up during the night (*p* = 0.023). Daytime fatigue decreased without changes in sleep time, which suggests improvements in both sleep quality and well-being. The findings also indicate the facilitation of the creation of a healthy positive sleep cycle (Sandberg et al., 2021).

#### 4.1.3 Dance in Combination With Traditional Physical Activity

A previous investigation examined combining a dance intervention with running activity using primary school students. The physical activity level, skinfolds reduction and endurance fitness showed the significant increases (*p* < 0.05) compared with a control group. For the secondary measurements, there were no change in dietary variables, knowledge, and majority of psychological indicators. However, the participants, teachers, and parents all responded positively. From the pupil’s perspective, most pupils enjoyed practicing dance and had a positive experience from joining the dance competitions. The parents all expressed that their children had a pleasant feeling from participating from the program, and because of their involvement, had become more aware of their own physical activity lifestyles (Morris et al., 2013). Dance also seems to have a positive effect on certain neuromuscular and neurovascular conditions.

Globally, 13.5% of school-aged children are affected by functional abdominal pain disorders (FAPDs). FAPDs include irritable bowel syndrome (IBS), functional dyspepsia, abdominal migraine, and functional abdominal pain (FAP). Abdominal pain is accompanied by other symptoms, such as depression, anxiety, reduced life quality, and school absenteeism (Högström et al., 2022). Previously, a research article demonstrated that Yoga had beneficial effects in reducing pain intensity, absenteeism, and IBS-related symptoms. Dance is a relaxed rhythmical activity, and when combined with yoga, seems to provide physical and mental benefits that reduce pain. In addition, dance is an extremely popular activity for young females. This research examined the benefits of dance and yoga on FAP using a female population. The 121 participants in the study were 9–13 years old girls who were diagnosed with FAP or IBS with persistent pain. The dance and yoga interventions were performed on two occasions per week lasting 8 months conducted during after-school courses. The key findings indicated that dance in association with yoga works better for this population than standard conventional health care methods for reducing maximum pain aspects. We can further hypothesize that these activities in combination might have been the strength of this intervention, as dance contributes to cardiorespiratory and rhythmic aspects of movement while yoga helps with focus, relaxation, and introspection (Högström et al., 2022). The socialization potential of the intervention may also have had positive impacts. Opportunities to engage with new friends and to observe other girls suffering from similar symptoms may have also helped facilitate the positive responses observed.

#### 4.1.4 Dance in Games

Over 60% of adolescents spend 73 min/day on video games (Staiano et al., 2017b). High levels of traditional and digital media use are linked to obesity, cardiovascular disease, and mental problems over the life course. These risks and associations have been observed to start in early childhood. Prolonged media use during preschool years is associated with increases in Body Mass Index (BMI). Body weight gain may be difficult to regress in combination with other risk factors, which increases the risk for greater weight gain and illness later in adult life (Robinson et al., 2010). This statement agrees with an international study that included almost three hundred thousand children and adolescents; the researchers found that watching TV 1–3 h per day led to a 10%–27% increase in obesity (Braithwaite et al., 2013).

As a result of the upsurge in computer use, some research studies have combined games and dance to cater for the characteristics of children and adolescents associated with media use and to minimize the effects of sedentary screen time. Dance-related computer games can increase the enjoyment and motivation of participation by allowing children and adolescents to take the initiative in selecting the variables of interest during the game. For example, participants can select the intensity levels, dance routines/mode, dance music, even dance game partners. In a research study investigating 36 h of dance exergaming lasting 12 weeks, researchers observed a decrease in adiposity and an increase in bone mineral density compared to a non-exercising control group (Staiano et al., 2017b). Furthermore, active video games (exergaming) facilitate exercise in a comfortable home environment, helps with exercise adherence and facilitates positive long-term changes in behavior. Recent studies have found exergaming to be far greater in enhancing energy expenditure when compared with non-active video games. The energy expenditure values obtained suggest that the intensities are comparable with moderate-intensity aerobic exercise (Wagener et al., 2012).

Active video game (exergaming) participation requires entire body movements. This results in light to moderate increases in energy expenditure and elevated heart rates. This could contribute to weight reduction and health benefits (Staiano et al., 2017a). In group settings, active video gaming may have benefits for increasing self-efficacy related to PA. There may also be beneficial effects for intrinsic motivation. Social cognitive theory suggests that behavioral change results from links among behaviors, the environment, and psychosocial variables (Staiano et al., 2017a). Group cohesion resulting from digital game play may be appealing to obese young people. These individuals are less likely to engage in traditional sports owing to excess weight, criticism, and bullying. Group active video play may provide a method of improving poor psychosocial health experienced by overweight and obese young people and facilitate increases in total PA levels (Staiano et al., 2017a). Future research is needed to investigate exergames and the design of dance games as enjoyable, sociable, motivating, and effective physical activity devices.

#### 4.1.5 Motor Development

Motor development defines physical growth and the strengthening of a child’s bones and muscles. It also defines an ability to move and touch his/her surroundings. For instance, if a child is good at gross motor skills such as crawling or walking, this affects cognitive development because he/she can easily move and explore their physical environment. In recent times, most children do not participate in PA outdoors; their favorite games no longer require large movements, and instead of using sports halls and open spaces, games are mostly played on cell phones, computers, or tablets (Anjos and Ferraro, 2018).

A randomized control study investigated a group who attended two classes of dance per week, over a 7-month period. The intervention was a specialized modified educational dance program. Using creative and ludic proposals, the intervention challenged the subjects to discover and experiment with new movement patterns and discover new ways of implementing the movements they already knew. The results of the study demonstrated significant improvements in motor development capabilities of the students exposed to educational dance lessons, compared with a control group. Both groups obtained positive results; however, the dance intervention group improved more. The improvements observed for motor skill development were maintained following cessation of the program. The author of the experiment stated that the practice of educational dance should be longitudinal as motor development is permanently evolving (Anjos and Ferraro, 2018).

### 4.2 Psychological Benefits of Dance

#### 4.2.1 Alleviation of Depressive Symptoms

A recent experiment focused on African-American girls aged 8–10 years old and their parents or guardians who were involved in a dance intervention lasting 2 years. Fasting total cholesterol levels, low-density lipoprotein cholesterol, and depressive symptoms decreased significantly among girls in the dance treatment group. There were no significant differences between groups for BMI (Robinson et al., 2010). A further study examined 12 weeks of dance movement therapy in adolescents with mild depression. The results suggested that dance movement therapy demonstrated positive improvements in the symptoms such as somatization, obsessive-compulsive disorder, interpersonal sensitivity, depression, anxiety, hostility, paranoid ideation, and psychoticism. All these variables are related to negative metal health problems (Jeong et al., 2005). Fatigue, stress, insomnia, and psychological symptoms are directly or indirectly linked to circulating levels of serotonin and dopamine. The increased plasma serotonin concentrations and decreased dopamine concentrations indicate possible therapeutic benefits for the decreases in depression observed in the dance movement therapy group (Jeong et al., 2005).

#### 4.2.2 Perceived Competence

Obese adolescents have sedentary existences and report feelings of embarrassment, fear of victimization and poor self-confidence about their ability to engage in exercise in group situations as powerful reasons for non-participation in physical activity (Wagener et al., 2012). In relation to this, a recent study considered a dance exergaming program in obese adolescents. The findings from the study indicated that the intervention group increased their perceived competence to participate in exercise from the start to the end of an exercise period compared with a control group (Wagener et al., 2012). Further benefits were that participants reported that there was an improvement in relationships with their parents. There was also a meaningful change in a high percentage of participants in the exergaming intervention that experienced improved internalizing and externalizing symptoms from baseline to the end of treatment compared to the control group. In addition, there was a very high adherence rate (98%) suggesting that group dance exergaming had a positive impact on improving obese adolescents’ self-efficacy to continue exercising and to cope with any perceived barriers to exercise (Wagener et al., 2012).

#### 4.2.3 Executive Function

Executive function plays a crucial role during childhood development. The developments include working memory capacity, inhibitory control, and cognitive flexibility (Rudd et al., 2021). Executive function is a particular area of interest during the developmental stages of early childhood and has been observed to be a superior indicator of academic achievement than IQ or socio-economic status (Oppici et al., 2020). Children with limited executive function are prone to a broad range of poor health and wellbeing outcomes in adulthood. Working memory is essential for understanding and making sense of new experiences as children develop over time. Low working memory capacity has been linked with poorer performance academically. As a result, designing suitable physical activity interventions that can improve working memory capacity in children are desirable and advantageous for children’s development. The improvements in executive function will eventually lead to a more intellectual and capable society (Oppici et al., 2020).

Dance is often accompanied by music to create a constant sense of pleasure and motor stimulation, that is, synchronized with performance. This also provides participants with many opportunities for whole-body movement. To investigate this, an RCT that included an 8-weeks intervention was administered to 6–7-year-old children to assess the efficacy of four executive function measures. The measures were working memory capacity, cognitive flexibility, inhibitory control, and motor competence. The interventions included two dance syllabuses. The results showed that both dance syllabuses improved inhibitory control ability. The choreographed syllabus also developed working memory capacity; unfortunately, the improvement of motor competence did not exceed normal development (Rudd et al., 2021).

A further study explored the effects of working memory capacity and motor competence in primary school children using different teaching pedagogies and different cognitive challenges; the experimental results showed no statistically significant differences between groups. However, the dance teachers added a cognitive challenge by limited visual presentations and encouraged children to use memories and recall movement sequences in the high-cognitive group. The results of the study demonstrated the possibility and suitability of using dance practice in combination with high cognitive challenges to improve working memory and motor competence in children. It also contributed to social skills development and the integration and enhancement of emotional elements resulting from performing in groups (Oppici et al., 2020). In addition to the benefits of dance enhancing executive function, dance has been shown to be advantageous in the development of convergent thinking. Convergent thinking is associated with the process of solving problems and finding a solution to a problem (Bollimbala et al., 2019). Recent studies have shown that a 20-min dance protocol as part of a regular 30-min physical education session contributed to an improvement in convergent thinking (irrespective of their BMI status). An RCT study did not establish a correlation between dance class and the development of creative potential. However, in terms of divergent thinking components (fluency and flexibility), participants with normal BMI showed improvements following a dance class intervention. The dance class group also demonstrated an increase in convergent thinking compared to the control group (Bollimbala et al., 2019).

#### 4.2.4 Internalizing Problems

Internalizing problems include depressed mood, low self-worth, and psychosomatic symptoms. Adolescent psychological health problems may have long-term negative effects on personal development; such as poor academic performance, social dysfunction, substance abuse, and suicide, especially in girls. Mental health problems have been cited to be some of the most alarming health issues and are estimated to affect 13% of children and adolescents globally. Female adolescents demonstrate a greater prevalence of health problems than their male counterparts. Females also experience greater levels of stress and somatic symptoms, and are more likely to experience pain and depression (Duberg et al., 2020). Results of an RCT demonstrated that a dance intervention significantly reduced somatic symptoms and emotional distress in adolescent girls after 8 months compared with traditional school health services (Duberg et al., 2020).

Another important study comprising adolescent girls aged 13–18 years old with internalizing problems who reported symptoms including pains in the head, stomach, neck, back, and/or shoulder, persistent feelings of tiredness, being worried, and being in low spirits, was completed using dance as the intervention. The intervention lasted 8 months, and self-rated health was measured using a single-item questionnaire which included general health, well-being, perceptions of symptoms, and vulnerability. The questionnaire has also been demonstrated to be both valid and reliable (Duberg et al., 2013). The dance intervention group improved their self-rated health far greater than the control group. The effects of the intervention remained for several months post intervention cessation. In addition, the results also demonstrated high adherence to the intervention and a positive experience for participants. This suggests that an intervention using dance is suitable for adolescent girls with internalizing problems (Duberg et al., 2013). The females participating in the study found the dance intervention to be enjoyable and undemanding, without any of the usual school pressures. The girls included had opportunities to provide input into the dance classes regarding the choice of music, and the girls participated in the creation of the choreography used. This may have created a sense of ownership for the participants, and the social developmental aspects are also important. The opportunity to make new friends and spend time participating in something they enjoy with others who have similar interests might be a powerful issue affecting recruitment, retention, and interest to participate (Duberg et al., 2013).

### 4.3 Medical Benefits of Dance

Down Syndrome (DS) is a congenital, genetic disorder caused by the presence of an extra partial or complete copy of chromosome 21. The neuromotor, musculoskeletal and cardiopulmonary systems are functionally problematic in children with DS and this impacts on their quality of life. Approximately fifty-eight percent of children with DS fail to meet the recommended 60 min of PA per day.

Traditional neuromuscular training lacks fun, creativity, and movement exploration. As an aesthetic movement art form, dance also has a positive psychotherapeutic impact, which may improve the intelligence and dual tasking of children with DS. In addition, children express their creativity and emotions such as joy, fun and happiness in the process of practicing and participating in dance, which provides children with body awareness, enthusiasm, and confidence. Ballet and Laban’s dance have been demonstrated to improve balance, rhythm, and autonomous control in children who were DS patients. A previous study used traditional Indian dance as an intervention investigating outcomes in 36 children with DS. Traditional Indian dance appeared to be beneficial for improving locomotor skills and balance capacity in children with DS. The intervention was more effective when compared with traditional neuromuscular training. There were no adverse movement effects or discomfort recorded during and following the dance sessions. These findings outline the safety and feasibility of Indian dance regimes for this group (Raghupathy et al., 2021).

In addition to the studies mentioned above, a further RCT investigating dance performance outcomes included children between the ages of 5 and 12 years, clinically diagnosed with spastic diplegic cerebral palsy (CP). The participants had Gross Motor Function Classification System (GMFCS) Scores of II/III. In this study, dance exercise was one of eight activities that all children were expected to perform. The study evaluated the efficacy, feasibility, and safety of home-based activity rehabilitation programs for children with diplegic CP. The results of the study and methodology used suggested that dance exercise was a good choice to be added to this activity package for diplegic CP sufferers (Cygan et al., 2020; Goswami et al., 2021).

### 4.4 Strengths and Limitations

To the best of our knowledge, this is the first systematic review investigating children and adolescent development using dance as an intervention. For inclusion in this review, each selected article was subjected to a peer review process prior to publication. In addition, each article had to present a clear, consistent methodology which added to research integrity.

Limitations of this review include that some of the articles only used females as participants. Therefore, generalizations about the study findings to male populations are difficult. Future studies should focus on the adaptations of dance interventions using both genders as participants. This will provide compelling evidence about the benefits of dance while minimizing the effects of gender specificity. A further limitation was that some of the studies outlined in this review used self-reported measurement tools. This may have introduced an element of recall bias. There was also a limited number of articles that were deemed suitable for inclusion based on the selection criteria.

## 5 Implications of Dance Exercise

Schools in many countries have traditionally hosted some form of health education program to develop knowledge, skills, and behaviors related to health awareness. Schools are in a unique position to provide healthy and academic outcomes *via* the implementation of health and wellness policies. Most children spend more time in school than any other location except for the home. Schools are crucial and practical for managing and providing information about childhood health risks. Because of the relationships between health status and the ability to learn, schools are in an exclusive location to influence healthy lifestyles for students by health policy implementation. Schools need to seriously consider this advantageous position to produce a solid healthy foundation in the growth stage of children that will have an important and positive impact on individuals, families, and society.

Dance and in particular creative dance, enriches the performance, composition, and appreciation of human movement, with a particular focus on producing aesthetic value. Dance performed in groups provides a social type of physical activity. Dance is also beneficial for increasing self-trust, self-esteem, and self-expression in children and adolescents (Duberg et al., 2020).

Students who engage in dance at school show greater initial socialization skills and better academic achievement compared with individuals who do not participate in dance. Dance internalizes the systems involved in art forms, and both children and adolescents can use the experience gained as tools for thinking, behaving, and regulating the inner world of their minds. Certain schools in Mainland China, provide dance programs as part of after-curriculum activities that are available on a weekly basis.

The findings reported here can be of value to practitioners, policymakers, and educational staff. Because of teaching experience and having witnessed the positive effects of providing students with a broad selection of physical activities, many teachers and practitioners support dance-based physical education (PE). Despite this, dance-based schemes remain vulnerable to exclusion from the PE curriculum. This is more likely in schools where PE is viewed as a developmental tool for the preparation for participation in competitive sport. Certain schools also view PE lessons as a medium to enhance and refine elite athletic performers. Further research is needed to examine if participation in dance enhances athletic performance, increases competitiveness, and is complementary to athletic development. The findings of this review could be interpreted as providing further evidence for the value of retaining and developing dance-based PE in the school curriculum. The findings also support the importance of dance in physical education provision more generally.

A consensus survey of PE teachers should be conducted to understand the views and feasibility of PE teachers regarding including dance as a part of PE curriculum and the implementation of dance for the existing curriculum and syllabus. There are also essential factors such as teacher training and curriculum development that need consideration. In the long term, overall improvements in health and physical fitness parameters result in improvements in the quality of life for individuals. Health policy amendments are needed to provide further support for the place of dance within the physical education curriculum.

## 6 Conclusion

In conclusion, dance develops relationships, connects people, and increases feelings of joy and togetherness. Dance has virtually no venue restrictions. It can be practiced at home, in isolation, in groups, or anywhere with suitable spaces. Dance requires no special equipment, and this characteristic is suitable for low-income families and financially limited regions and countries. In summary, dance can be used as an appropriate and alternative physical activity mode for children and adolescents. The implementation of dance programs needs serious consideration by policy makers, schools, guardians and parents to produce greater long-term increases in physical activity in the foreseeable future. We hope that this systematic review will stimulate debate and provide more evidence for governments, schools, parents, and associated community officials to attach importance to dance as a medium of physical activity. Comprehensive and integrated changes are needed in relation to school/family/government/community partnerships. These changes include political and financial support from policy makers, and increased dance evaluation research that are important for a physical activity health policy reconfiguration and subsequent implementation.

## Data Availability

The original contributions presented in the study are included in the article/supplementary material, further inquiries can be directed to the corresponding author.
